# Micro-nanoplastics and cardiovascular diseases: evidence and perspectives

**DOI:** 10.1093/eurheartj/ehae552

**Published:** 2024-09-06

**Authors:** Francesco Prattichizzo, Antonio Ceriello, Valeria Pellegrini, Rosalba La Grotta, Laura Graciotti, Fabiola Olivieri, Pasquale Paolisso, Bruno D’Agostino, Pasquale Iovino, Maria Luisa Balestrieri, Sanjay Rajagopalan, Philip J Landrigan, Raffaele Marfella, Giuseppe Paolisso

**Affiliations:** IRCCS MultiMedica, Polo Scientifico e Tecnologico, Via Fantoli 16/15, 20138 Milan, Italy; IRCCS MultiMedica, Polo Scientifico e Tecnologico, Via Fantoli 16/15, 20138 Milan, Italy; IRCCS MultiMedica, Polo Scientifico e Tecnologico, Via Fantoli 16/15, 20138 Milan, Italy; IRCCS MultiMedica, Polo Scientifico e Tecnologico, Via Fantoli 16/15, 20138 Milan, Italy; Section of Experimental and Technical Sciences, Department of Biomedical Sciences and Public Health, School of Medicine, Università Politecnica delle Marche, Ancona, Italy; Department of Clinical and Molecular Sciences, Disclimo, Università Politecnica delle Marche, Ancona, Italy; Clinic of Laboratory and Precision Medicine, IRCCS INRCA, Ancona, Italy; Department of University Cardiology, IRCCS Galeazzi-Sant'Ambrogio Hospital, Milan, Italy; Department of Environmental Biological and Pharmaceutical Sciences and Technologies, University of Campania ‘Luigi Vanvitelli’, Caserta, Italy; Department of Environmental Biological and Pharmaceutical Sciences and Technologies, University of Campania ‘Luigi Vanvitelli’, Caserta, Italy; Department of Precision Medicine, The University of Campania ‘Luigi Vanvitelli’, Naples, Italy; University Hospitals, Case Western Reserve University School of Medicine, Cleveland, OH, USA; Program for Global Public Health and the Common Good, Boston College, Chestnut Hill, MA, USA; Centre Scientifique de Monaco, Monaco, Monaco; Department of Advanced Medical and Surgical Sciences, University of Campania ‘Luigi Vanvitelli’, Naples, Italy; Department of Advanced Medical and Surgical Sciences, University of Campania ‘Luigi Vanvitelli’, Naples, Italy; UniCamillus International Medical University, Rome, Italy

**Keywords:** Plastics, Pollution, Cardiovascular events, Heart disease, Environmental, Exposome

## Abstract

Emerging evidence indicates that chemical exposures in the environment are overlooked drivers of cardiovascular diseases (CVD). Recent evidence suggests that micro- and nanoplastic (MNP) particles derived largely from the chemical or mechanical degradation of plastics might represent a novel CVD risk factor. Experimental data in preclinical models suggest that MNPs can foster oxidative stress, platelet aggregation, cell senescence, and inflammatory responses in endothelial and immune cells while promoting a range of cardiovascular and metabolic alterations that can lead to disease and premature death. In humans, MNPs derived from various plastics, including polyethylene and polyvinylchloride, have been detected in atherosclerotic plaques and other cardiovascular tissues, including pericardia, epicardial adipose tissues, pericardial adipose tissues, myocardia, and left atrial appendages. MNPs have measurable levels within thrombi and seem to accumulate preferentially within areas of vascular lesions. Their presence within carotid plaques is associated with subsequent increased incidence of cardiovascular events. To further investigate the possible causal role of MNPs in CVD, future studies should focus on large, prospective cohorts assessing the exposure of individuals to plastic-related pollution, the possible routes of absorption, the existence of a putative safety limit, the correspondence between exposure and accumulation in tissues, the timing between accumulation and CVD development, and the pathophysiological mechanisms instigated by pertinent concentrations of MNPs. Data from such studies would allow the design of preventive, or even therapeutic, strategies. Meanwhile, existing evidence suggests that reducing plastic production and use will produce benefits for the environment and for human health. This goal could be achieved through the UN Global Plastics Treaty that is currently in negotiation.

## Graphical Abstract

**Graphical Abstract:**
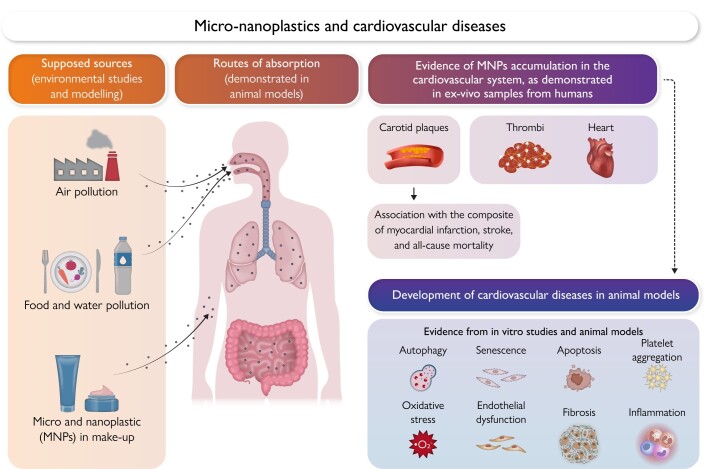
Summary of the available knowledge relative to the possible role of micro- and nanoplastics (MNPs) in cardiovascular disease. Environmental and modelling studies suggest that air (both indoor and outdoor), water (both bottled and tap water), food, and cosmetic products are possible sources of exposures to MNPs in humans. Such MNPs can be absorbed through inhalation, ingestion, or even skin contacts, as suggested by animal models. Once reached the bloodstream, such MNPs might accumulate in multiple cardiovascular tissues, as evidenced by studies employing *ex-vivo* samples from humans. Here, they might promote the activation of a number of deleterious pathways promoting low-grade inflammation, oxidative stress, endothelial dysfunction, alteration of autophagy, and apoptosis, as proposed by experiments *in vitro* and in animal models. In humans, the presence of MNPs in carotid plaques is associated with the subsequent incidence of a composite of myocardial infarction, stroke, and all-cause mortality.

## Introduction

The discovery of petroleum-derived plastics has transformed the industrial landscape, permeating every facet of manufacturing and consumption. Their low cost and ease of production have made plastic polymers the predominant materials for a wide range of applications, from food packaging to construction.^1^ However, a reconsideration of plastics’ ecological repercussions and sustainability considerations is gradually limiting their unfettered use and has prompted the United Nations Environment Assembly to call for development of a Global Plastics Treaty.^2^

Beyond the well-established environmental threat associated with plastic-related pollution, there is need to deepen understanding of the possible consequences on human health of widespread use of plastics.^3^ While there are already warnings that plasticizers and other plastic-associated chemicals, such as Bisphenol A and phthalates, promote a range of adverse health outcomes through their endocrine-disrupting properties and other mechanisms,^4^ recent evidence suggests a possible deleterious role for micro- and nanoplastics (MNPs).

Microplastics and nanoplastics are plastic particles with sizes below 5 and 1 µm, respectively. MNPs can be primary, e.g. manufactured MNPs deliberately added to products, such as cosmetics, or secondary byproducts of the chemical and/or mechanical fragmentation of plastic-related waste.^5,6^ MNPs have become widespread throughout the Earth's biosphere and are detectable in the air, water, food, and drinking water.^2,7,8^ Animal models suggest that MNPs might be absorbed through ingestion, inhalation, or even through skin contact, and that they trigger a range of possible adverse health effects.^9^ Recent estimates suggest that humans might inhale or ingest millions of MNP fragments via these routes during their life.^10^ Accumulating evidence suggests that MNPs accumulate in different tissues in humans.^11^

Among the range of possible effects on human health, MNPs are emerging as a possible risk factor for the development of cardiovascular diseases (CVD). Findings from *in vitro* studies advance the hypothesis that MNPs trigger a range of pathophysiological pathways in cells relevant to the development of CVD. These involve pathways through endothelial and immune cells and involve pathophysiologic alterations that include endothelial dysfunction and immune activation. These findings are corroborated by animal models that suggest such alterations following treatment with MNPs.^12^ Preliminary evidence from humans substantiates the accumulation of and a possible pathological role of MNPs in the cardiovascular system.^12–15^

Here, we briefly summarize the major mechanistic studies linking MNPs to CVD in preclinical models to then synthesize the data showing MNP accumulation in humans. We focus on studies reporting the detection of MNPs in cardiovascular tissues, discussing the reported association with CVD and related phenotypes. Finally, we suggest the need for future studies to further elucidate the possibility that MNPs may be a novel risk factor for CVD.

## *In vitro* effects of MNPs on endothelial and immune cells

A number of studies have explored the impact of different MNPs on endothelial cells, immune cells, and other cell types relevant for the pathogenesis of CVD.^12^ Given the initial lack of data relative to the accumulation of MNPs in humans, pioneering *in vitro* experiments have focused largely on MNP types with the highest likelihood of contact with humans, e.g. polystyrene.^12^ However, a range of MNP sizes, doses, and shapes have been tested (summarized in Supplementary data online, *Table S1*).

Experiments with labelled MNPs or other techniques suggest their ability to enter different cell types. Indeed, following *in vitro* treatment, MNPs are not only detectable within cells with a known phagocytic activity, e.g. macrophages, but also in endothelial and other cells, possibly due to the ability of MNPs to disrupt membrane properties.^16–19^ Once they are inside, cells unsuccessfully try to digest MNPs, engulfing their lysosomes.^18–21^ In the case of monocytes/macrophages, MNP accumulation in the cytoplasm is paralleled by the accumulation of lipid droplets, a known step in the formation of foam cells.^20^ With the goal of eliminating the foreign particle, immune cells activate NADPH-oxidase and other enzymes to produce reactive oxygen species, e.g. superoxide and hydrogen peroxide, which in turn foster a pro-oxidant cascade.^22^ Of interest, MNPs might also generate free radicals, even before contact with living cells, due to the effect of photo oxidation or UV light radiation in the environment, even though the health relevance of this phenomenon is unknown.^23^ The induction of oxidative stress by MNPs is common in multiple cell types, including endothelial cells,^24^ and not confined to specialized phagocytes.

Parallel and/or subsequent to oxidative stress, MNPs foster inflammatory responses in multiple cell types. Indeed, different MNPs can promote the activation of the NLRP3 inflammasome, of NF-κB, and of other major pro-inflammatory pathways.^25–28^ Of note, data suggest that phagocytosis might not be necessary to induce such phenomena, possibly extending the detrimental effect of MNPs also beyond a certain size, intuitively required for ingestion by cells.^29,30^ However, in case of internalization, the activation of the innate immune system is an obvious consequence of such event. Indeed, crystalline silica, metals, asbestos, and other exogenous as well as insoluble endogenous particles, e.g. urate or cholesterol crystals, are all well-established triggers of sterile, chronic, low-grade inflammation.^29,31^

Another key phenomenon possibly linking MNPs to low-grade inflammation is cellular senescence, which is defined as a permanent state of cell cycle arrest coupled by the secretion of pro-inflammatory and other factors.^32^ A number of reports evidenced an increase in rates of senescence in multiple cell types, including endothelial cells, after treatment with different types of MNPs.^33–35^ Cellular senescence, similar to oxidative stress and the inflammasome, is increasingly becoming a therapeutic target for drug development. Selected compounds promote the clearance of senescent cells while others prevent their formation or suppress their noxious pro-inflammatory program.^36^ One report suggests that sodium–glucose cotransporter-2 (SGLT-2) inhibitors, a class of glucose-lowering drugs, can attenuate the senescence induced by MNPs, which increases the expression of this transporter in the membrane of endothelial cells.^34^ Of note, SGLT-2 inhibitors have demonstrated cardioprotective properties in multiple contexts.^37–39^

Experiments using whole blood evidenced a range of effects associated with MNP treatment, including the induction of platelet aggregation, hemolysis, and immune cell activation.^16,40–42^ However, most of this *in vitro* work used unrealistic doses of MNPs possibly not relevant to emerging pharmacokinetic evidence from human samples (see below). Similarly, most of the MNP types tested were not necessarily those found in tissues in later work or were of different sizes (see Supplementary data online, *Table S1*). These aspects might limit the mechanistic relevance of these findings, especially considering that the chemical properties and the size of MNPs influence their absorption, distribution, internalization by cells, and ability to promote deleterious pathways.^43–48^ Preliminary data suggest that positively charged MNPs might be especially harmful, particularly in regards to platelet aggregation.^49^

In summary, the available *in vitro* evidence suggests that MNPs can enter human cells and foster a large range of pathophysiologic pathways and mechanisms previously associated with CVD development, i.e. oxidative stress, cellular senescence, platelet aggregation, and especially low-grade inflammation. If confirmed by preclinical studies employing pertinent dosages and types of MNPs, these phenomena might represent possible points of intervention to limiting the damage induced by MNP accumulation.

## MNP absorption and cardiovascular effects in experimental models

Data from animal models suggest the possibility that all the three main routes of entry—inhalation, ingestion, and even skin contact—can allow MNPs to be absorbed into the body.^11,50,51^ Particle size influences the ability of MNPs to reach multiple tissues, and the absorption and distribution of MNPs increase as particles size decreases.^45,52,53^ Similarly, the physicochemical features of different MNPs affect their ability to reach distant organs, with negatively charged MNPs being characterized by a higher degree of distribution.^47^ Of note, highly vascularized organs and blood vessels seem to preferentially accumulate MNPs.^12,54^

While a number of reports suggest that MNPs alter the development of the cardiovascular system in organisms, such as zebrafish and other fishes,^12^ fewer data are available relative to the cardiac effects of MNPs in rodents (summarized in *Table 1*). Short-term, oral exposure to polystyrene MNPs in mice and rats results in accumulations in the blood and the heart, with detectable levels in isolated cardiomyocytes.^45,47,57,67^ MNP treatment is associated with a wide range of deleterious effects, including cardiac fibrosis, capillary hyperemia or congestion, thinner or ruptured myocardia, myocardial fibre breakage, myocardial inflammatory injury or apoptosis, and the subsequent elevation of cardiac enzymes.^57–59^ These phenotypes are promoted by the activation of the Wnt/β-catenin and the NLRP3/caspase-1 signaling pathways, as well as by interference with electrical synchronization.^57–59^ The ability of MNPs to alter the cardiac structure was also confirmed in human-derived organoids.^63^ At the microvascular level, treatment with MNPs in mice or rats is associated with an increase of pro-thrombotic phenomena after stimulation.^49,55,56^

**Table 1:** Summary of the studies assessing the effect of micro-nanoplastics in animal models of cardiovascular diseases

Study	Animal model	Micro-nanoplastic type (and size)	Dose	Treatment	Condition studied	Effects observed
Silva et al. (2005)^55^	Fischer 344 rats	PS (60 nm)	Amine-PS-NPs at 0.02, 0.5, and 50 mg/kg, carboxylate-PS-NPs at 0.1 and 50 mg/kg	Intravascular or intra-tracheal administration	Thrombosis	Amine-coated PS MNPs promoted thrombosis
Bihari et al. (2010)^56^	C57BL/6NCrl mice	PS (60 nm)	0.5 mg/kg body weight	Intravenous injection, 10 min prior to the ferric chloride-induced thrombosis	Platelet activation	Amine-coated PS MNPs shortened the occlusion time of mesenteric arteries, enhanced P-selectin expression, and promoted the formation of platelet–granulocyte complexes. Carboxylate-coated PS MNPS lengthen the occlusion time of mesenteric arteries
Smyth et al. (2015)^49^	C57BL/6 mice	PS (50 nm)	1.2 µg	Injection, followed by 50 µg/kg of collagen	Platelet aggregation	Amine-coated MNPs enhanced platelet aggregation when collagen is administered after treatment
Li et al. (2020)^57^	Wistar rats, male	PS (0.5 µm)	0.5, 5, and 50 mg/L	Oral for 90 days	Cardiomyocyte apoptosis	Capillary hyperaemia, thinner or breakage myocardia, mitochondrial cristae disappear, and upregulation of CK-MB and cTnI
Roshanzadeh et al. (2021)^58^	Neonatal rat ventricular cardiomyocytes	PS (50 nm)	25 µg/mL	Exposition to electrical pulses, for 60 min	Cardiac contraction	Impairment of contractile function of neonatal rat ventricular myocytes treated with amine-coated PS-MNPs
Wei et al. (2021)^59^	Wistar rats, male	PS (0.5 mm)	0.5, 5, and 50 mg/L	Oral for 90 days	NLRP3/Caspase-1 signaling pathway and oxidative stress	Capillary congestion, myocardial fibre rupture, and upregulation of CK-MB and cTnI
Vlacil et al. (2021)^25^	C57BL/6N mice	PS (1 µm)	2.5 mg	Intravenous injection for 3 h	Endothelial inflammation	Carboxylate-PS-MNPs enhanced IL1β and Icam-1 expression in aortic tissue
Zhao et al. (2022)^60^	C57BL/6 mice, male	PS (0.5 and 5 µm)	0.1μg/mL and 1μg/mL	Oral for 12 weeks	Adipogenesis-related and inflammation-related pathways	MNPs promoted the onset of cardiometabolic diseases
Yan et al. (2023)^61^	Sprague–Dawley (SD) rats, male, 7–8 weeks old	PS (5 μm)	0.5 mg/L	Exposure for 120 days	Inflammatory pathway	Exposure to PS-MNPs caused mild vascular calcification in healthy rats and worsened vascular calcification in rats treated with vitamin D3 and nicotine
Zhang et al. (2023)^62^	C57BL/6J mice, male, 6–8 weeks	PS (40 nm)	0 µg/day, 16 µg/day, 40 µg/day and 100 µg/day	Inhalation for three exposure durations (1 week, 4 weeks, 12 weeks)	Mitochondria damage, cardiotoxicity	MNPs caused cardiac structural and functional damage in a dose-and time-dependent manner
Zhou et al. (2023)^63^	Specific pathogen-free (SPF) male Balb/c mice	PS (1 μm)	0.025, 0.25 and 2.5 µg/mL	Instilled intra-tracheally twice a week for 4 weeks	Oxidative stress, inflammatory response, apoptosis, and collagen accumulation.	MNPs induced cardiac hypertrophy both *in vivo* and *in vitro* experiments
Zhang et al. (2023)^64^	Male, 6 week-old C57BL/6 mice	PS (10 µm)	1000 g/L	Drinking water for 180 days	Endocytosis, cellular senescence, and cell cycle signaling pathways	MNPs caused an alteration of lncRNAs and circRNAs and promoted cardiotoxicity
Wang et al. (2023)^65^	ApoE−/−mice	PS (50 nm)	2.5–250 mg/kg	Oral gavage with a high-fat diet for 19 weeks	Phagocytosis of M1-macrophage in the aorta and lipid metabolism	MNPs in the blood and aorta of mice worsened artery stiffness and lead to the formation of atherosclerotic plaques
Zhang et al. (2024)^66^	C57BL/6 mice (6 week-old males)	PS (100 nm)	10–100 μg/mL	Exposure for 30 or 180 days	tiRNA-Glu-CTC/Cacna1f axis.	MNPs exposure induced vascular injury. 50 μg/mL of PS MNPs induces vascular smooth muscle cell (VSMC) phenotypic switching, whereas 100 μg/mL triggers VSMC apoptosis
Zhang et al. (2024)^54^	8 week-old male C57BL/6 mice	Red fluorescent PS (100 nm)	100 μg/mL	Drinking water for 30 or 180 days	MAPK signaling	Vascular toxicity with changes in lipid processing and thickening of the artery wall

Mechanistically, acute exposure to MNPs evokes consistent inflammatory and immune responses.^62^ Indeed, treatment with polystyrene MNPs promotes endothelial inflammation, as evidenced by an increased expression of interleukin (IL)-1β and intercellular adhesion molecule-1 in the aortic tissue^25^ while also enhancing enhanced aortic sensitivity to phenylephrine.^60^ Of note, the induction of chronic, systemic pro-inflammatory responses by MNPs involves also the adipose tissue, a phenomenon that might occur also independently of the absorption of particles. Indeed, administration of polystyrene MNPs through drinking water in mice induce weight gain and an increased expression of IL-6 and monocyte chemoattractant protein-1 in the perivascular adipose tissue, an effect paralleled by a large derangement of the microbiome.^60^

Multiple reports have confirmed pro-atherosclerotic effects of MNPs in animal models. Indeed, polystyrene MNPs, gavage-fed to high-fat diet-fed mice promotes arterial stiffness and atherosclerotic plaque formation. MNPs activate phagocytosis of M1-macrophages, disrupting lipid metabolism, and fostering foam cell accumulation.^65^ In addition, another study suggested that the vascular injury induced by chronic exposure to low-dose polystyrene MNPs could be mediated by their ability to induce vascular smooth muscle cell phenotypic switch.^66^ Whatever the mechanism, experiments with labelled MNPs clearly suggest that their uptake results in vascular toxicity and the thickening of the arterial wall.^54^

In summary, similar to the *in vitro* work, most of animal experiments have employed polystyrene MNPs, preferentially in acute settings, with a high dosage of particles administered through ingestion. This aspect may limit the human relevance of such findings. In addition, it impedes drawing firm conclusions relative to which MNP type is more harmful, given the lack of comparative studies. However, even chronic, low-dose administration of MNPs in mice and rats is associated with a range of cardiovascular alterations, including direct cardiac damage and a pervasive pro-atherosclerotic effect (*Table 1*). Whether there is a clear threshold in terms of dosage and/or duration of exposure that is required to exert deleterious effect has not been thoroughly explored.

## Evidence of MNP accumulation in humans

A consistent burden of evidence documents the widespread presence of MNPs across diverse environmental domains, such as surface water, sediment, wastewater, sea ice, indoor and outdoor air, bottled and tap water, and multiple foods.^11,68,69^ A recent estimate suggests that 86 to 710 trillion MNP particles contaminate European agricultural land each year,^70^ with virtually all MNP types being detectable in this context.^71^ The discovery of MNPs in seafood, honey, milk, beer, table salt, drinking water, and airborne particles is now spurring the study of the potential impacts of these particles on human health.^11,72,73^ A mathematical model suggests a staggering per capita intake of 74 000–121 000 MNPs annually through the consumption of food, water, and dust, and inhalation of air.^72^ Another model estimated the yearly intake to range from 39 000 to 52 000 items per person . This included contributions from various sources, such as 37–1000 MNPs from sea salt, 4000 from tap water, and 11 000 from shellfish.^72^ Further insights emerged from a probabilistic lifetime exposure model, which projected MNP intake rates of 184 ng/capita/day for children and 583 ng/capita/day for adults across nine different possible exposures.^74^ A systematic review of articles assessing possible exposure from multiple sources estimated a yearly mass-based intake ranging from 15 to 287 g per person,^75^ highlighting the multifaceted nature and potentially large scale of human exposure to MNPs.

Given this potentially broad exposure, studies have investigated evidence of MNP accumulation in human tissues. The technologies used for detection in many of these studies are not uniform. Raman spectroscopy, Fourier transform infrared (μFTIR) micro-spectroscopy and laser-directed infrared (LD-IR) estimate the MNP size and the relative number in the sample analysed, but do not provide the effective concentrations of the compounds detected in terms of weight of selected MNP/weight of the tissue. In contrast, pyrolysis–gas chromatography–mass spectrometry (Py–GC/MS) furnishes an estimate of the concentration of different plastic types, but without information relative to MNP size and number. Given these limitations, information collected from different tissues with diverse technologies are not standardized and might not always be comparable.

Supplementary data online, *Figure S1* summarizes all the evidence relative to the detection of MNPs in the human tissue with the exception of the cardiovascular system. Evidence of the presence of MNPs has been provided in samples from multiple human tissues or biological fluids, such as the placenta,^76–79^ lung,^80–82^ liver,^83^ breastmilk,^84^ urine,^85^ sputum,^86^ stool or meconium,^77,78,87–89^ blood,^90^ kidney,^91^ colon,^92^ semen/testis,^93^ and the endometrium.^94^ Thus, virtually every human organ may accumulate some forms of MNPs. Relative to their size, MNPs of up to 30 µm in size have been detected in the liver, up to 10 µm in placenta, up to 88 μm within lungs, up to 10–15 µm in breastmilk, urine, and the kidney, up to 500 μm in the endometrium, and even larger particles in the colon (see Supplementary data online, *Figure S1*). The evidence relative to the lungs and the colon might suggest that both inhalation and the ingestion are possible routes of MNP absorption. In addition, they might highlight that, at least in selected subjects, the proposed threshold of MNPs size for their entrance, i.e. 150 μm, might not always be respected.^52^ By contrast, most of the MNPs detected in difference tissues were below 10 μm in diameter, supporting a higher degree of absorption and/or distribution for smaller particles. Of note, one study suggested a possible gender-related difference in MNP accumulation, with women showing a higher abundance of detectable MNPs in samples from tonsils, lungs, and the intestine.^95^ Possible phenomena explaining this observation might be a higher exposure to MNPs in women or simply a difference relative to body structure or weight.

The same studies identified more than 10 different types of polymers in human tissues. Among others, those most commonly identified were polyethylene (PE), polyvinyl chloride (PVC), polyethylene terephthalate (PET), polypropylene (PP), and polystyrene (PS) (see Supplementary data online, *Figure S1*). This might be attributable to the fact that these compounds are those more commonly tested. Alternatively, these molecules may be more often detectable since they are those with the widest range of application in everyday life and are found in animal species as well as humans. Indeed, most food, liquid, or cosmetic containers, as well as water pipes, are made of these plastics, rendering hard to distinguish and quantify the contributions from multiple, diverse sources of potential exposure.

Most of the studies providing evidence of the presence of MNPs in different organs have not found evidence for a link or association with a pathological phenotype. Thus, the available evidence is insufficient to posit a clear, general pathogenic role for MNPs at present. With the exception of CVD (see below), only two studies found a cross-sectional association between MNPs presence and a disease. Indeed, MNPs were detectable in patients with cirrhotic disease, but not in healthy livers,^83^ while the abundance of MNPs in stool samples was higher in patients with inflammatory bowel disease compared with subjects without this condition.^89^ Thus, more studies with clinical data and especially longitudinally collected hard endpoints are necessary to sustain a broad pathogenic role for MNPs.

## MNPs in the human cardiovascular system

**Figure 1:**
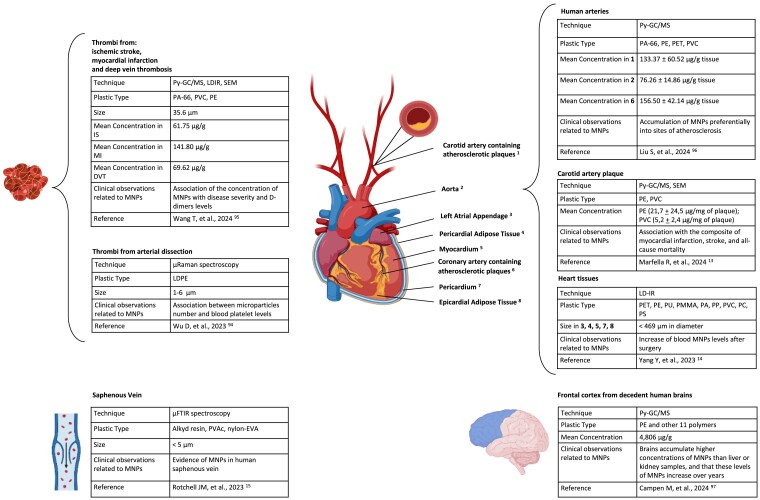
Evidence of micro- and nano-plastic (MNP) accumulation in the human cardiovascular system and the brain. Summary of the evidence relative to the presence of different MNPs in human samples from the cardiovascular system, along with the technology used for their detection, the size or the concentrations of the different MNPs, and the clinical observations associated with the presence of MNPs. List of acronyms: PC, polycarbonate; μ-FTIR, micro Fourier transform interferometer spectroscopy; LDPE, low-density polyethylene; PET, polyethylene terephthalate; PP, polypropylene; PS, polystyrene; PU, polyurethane; PVC, polyvinyl chloride; LD-IR, laser direct infrared spectroscopy; PA, polyamide; PVA, polyvinyl alcohol; PVAc, polyvinyl acetate; PMMA, polymethyl methacrylate; Py–GC/MS, pyrolysis–gas chromatography–mass spectrometry; nylon-EVA, nylon ethylene-vinyl acetate; SEM, scanning electron microscopy

The cardiovascular system, and in particular the endothelium, is exposed to all of the substances present into the bloodstream. Given that MNPs are small enough to be absorbed and be detectable in blood, it is conceivable that such particles can also penetrate blood vessels. At least five reports have documented the presence of at least one type of MNPs in *ex-vivo* samples derived from the cardiovascular system (summarized in *Figure 1*).

A preliminary study analyzing five human saphenous vein tissue samples using μFTIR spectroscopy (size limit of 5 μm) reported not only a high background contamination, but also detectable levels of MNP types not present in blank samples. MNPs were mostly of irregular shapes, and five different polymers were detected.^15^ Another study explored the presence of MNPs in 15 patients undergoing cardiac surgery, assessing the presence of particles through LD-IR and SEM in pericardia, epicardial adipose tissues, pericardial adipose tissues, myocardia, left atrial appendages, and pairs of pre- and post-operative venous blood samples. This study found a range of nine different MNP types in both cardiac tissues and blood samples, with particle sizes up to 469 μm in diameter. However, the size of most of the MNPs was <50 μm. Technically, the LD-IR system does not identify MNPs with a diameter smaller than 20 μm, impeding an estimate of the abundance of MNPs in the very small range (which should have a higher degree of distribution). By contrast, the authors confirmed the presence of poly(methyl methacrylate) in the left atrial appendage, epicardial adipose tissue, and pericardial adipose tissue that could not be attributed to accidental exposure during surgery, thus sustaining the possible accumulation of MNPs in the heart.^14^

Two studies assessed the presence of MNPs within thrombi collected from different vascular regions. While a preliminary study employing Raman Spectrometer found evidence of low-density PE particles below 6 μm,^96^ a recent manuscript found evidence for a range of different MNPs in thrombi from a heterogeneous cohort of 30 patients undergoing thrombectomy due to ischemic stroke, myocardial infarction, or deep vein thrombosis.^97^ According to Py–GC/MS, 24 of 30 thrombi assessed had detectable levels of MNPs and in particular of polyamide 66 (PA66), PVC, and PE. The latter was the most abundant being present in more than half of the samples, with a mean diameter of 35.6 μm. The shapes of MNPs according to LDIR and SEM were heterogeneous. Of note, concentration of MNPs was associated with disease severity, while the level of D-dimer was higher in patients with evidence of MNPs compared with those without, suggesting a cross-sectional association with the severity of CVD.^97^

Two independent studies evaluated the presence of MNPs into samples derived from atherosclerotic plaques and provided data that were somewhat discordant yet compatible with the evidence previously collected from thrombi. One study quantified 10 types of MNPs through Py–GC/MS in plaque specimens obtained from carotid or coronary arteries, as well as samples from aortas.^98^ All these three sample types had detectable levels of PA66, PVC, PE, and PET, with the latter being the most abundant. Of note, the concentration of MNPs in arteries containing atherosclerotic plaques, both coronary and carotid arteries, was significantly higher than that in aortas, which did not contain atherosclerotic plaques, suggesting that MNPs might accumulate preferentially into sites of atherosclerosis.^98^ Another study used the same technology to quantify MNPs specifically in carotid plaques excised from 257 patients undergoing carotid endarterectomy and followed-up for 3 years to monitor the incidence of myocardial infarction, stroke, and all-cause mortality, which represented the primary endpoint.^13^ Among these, 150 patients had evidence of PE within the plaque, whereas 32 of these also had measurable amounts of PVC. Analysis with electron microscopy revealed the appearance of ‘jagged-edged particles’ both among plaque macrophages and scattered in the external debris. Patients with evidence of MNPs had a higher expression of inflammatory markers, i.e. IL-18, IL-1β, tumor necrosis factor-α, IL-6, CD68, and CD3, and a lower abundance of collagen within the plaque. More importantly, patients in whom MNPs were detected within the atheroma were at higher risk for a primary end-point event than those in whom these substances were not detected, a finding that represents the first evidence of prospective association between MNPs and an hard health outcome and in particular CVD.^13^

Evidence relative to accumulation of MNPs in brain vessels has not yet been provided. However, a recent manuscript reported evidence for the presence of MNPs in brain samples derived from autopsies, suggesting that brains may accumulate higher concentrations of MNPs compared with liver or kidney samples, and that such levels of MNPs increased during years.^99^ Associations of MNPs with specific phenotypes were not explored.

Beyond the direct evidence of MNP accumulation in the cardiovascular system, one study explored the possibility of an indirect association between the MNP delivery to the intestine and CVD. In 47 patients in a study distinguished by the presence of absence of calcification in the thoracic aorta wall, the presence of MNPs in stool samples from this population through μFTIR was evaluated. Patients with vascular calcification had higher levels of total MNPs, PP, and PS in feces than patients without this condition, while the thoracic aortic calcification score was positively correlated with MNP levels.^61^ These results could support the hypothesis, described above in animal models, that MNPs do not need to be absorbed to promote deleterious effect on the cardiovascular system. Alternatively, they could simply reflect a higher exposure to MNPs in patients with CVD.

Overall, these data highlight that MNPs comprised various polymers, and plastic additives have been identified in thrombi, atherosclerotic plaques, and other cardiovascular tissues from humans. MNPs might have a propensity to accumulate within regions with vascular lesions, and their presence in the carotid plaque correlates with a subsequent heightened risk of cardiovascular events or mortality.

## Open questions and future research

Every stage of the plastic life cycle, from extraction of the coal, oil, and gas that are its main feedstock through to their ultimate disposal into the environment, is detrimental to the environment and potentially harmful for human health. The extent and the magnitude of the issue, as well as its economic cost, have only partially been explored.^3^ Geographical variations are influenced by economic, anthropogenic, and cultural factors and environmental conditions. The lack of consistency and standardization of sampling and analytical methods for detection of MNP pollution inhibits a global comparison of MNP deposition.^5^ Increasing efforts are required to better comprehend the sources, pathways, and impacts of MNPs on ecosystems and human health.^100^ These include studying the long-term effects of exposure to MNPs, identifying emerging sources of pollution, and developing specific and sensitive methods for detecting and quantifying MNPs in different environmental and biological matrices. In addition, the technology used to detect MNPs should be standardized. Innovative or repurposed approaches to chemically detect and quantify MNP *in vivo* and the putative development of biomarkers of toxicity in human biological samples, such as blood or saliva, remain major issues that need to be addressed.

The relationship between exposure and accumulation of MNPs in human tissues is a critical issue in assessing causation of the health effects of plastic pollution. There is little evidence, for example, of an association between lifestyle choices and the accumulation of MNPs in human tissues. A relevant issue is to assess what types of exposure, inhalation, ingestion, or dermal exposure are most relevant for cardiovascular health. To get as these questions, a key variable is to estimate the dose of exposure. At present, there is no validated instrument, e.g. a structured questionnaire, to assess the exposure to plastic-related pollution. Such an instrument, coupled by the temporal relationship between exposure and accumulation in tissues, would facilitate the design of long-term, prospective studies linking exposure, absorption, and accumulation to the incidence of hard outcomes, including CVD, favoring also the study of the existence of a putative safety limit.

Several molecular mechanisms might both facilitate tissue uptake of MNPs and increase their pathogenicity. Also, MNPs have potential to act as potential transporters of contaminants and as chemosensitizers for other toxic substances. Following exposure, bioavailable particles that enter the circulatory system can translocate to secondary organs, where they might accumulate to a level that could result in adverse effects at the cellular level. However, there are currently many open questions regarding how plastic particles of different sizes are distributed in the body, including the localization in specific cells, such as those of the immune system. A comprehensive approach to understand the immunotoxic effects of MNPs and their immunogenicity is warranted. The mechanisms of MNP adhesion and uptake and their accumulation should also be extensively investigated.

The evidence collected to date relative to MNPs and CVD is associative and derives from patients with manifest CVD. Thus, no cause-effect relationship can be considered established at this stage. For instance, relative to the association of MNPs within the carotid plaque and CVD and given that MNPs seem to accumulate within plaque macrophages,^13^ it is unknown whether MNP accumulation precedes or follows macrophage accrual. Indeed, it is possible that patients with a poor plaque phenotype and thus with a more consistent immune infiltrate has a greater tendency to uptake MNPs. Alternatively, it is possible that MNPs promote a systemic and/or a local inflammatory response, fostering the development and the instability of plaques. To sustain causality, long-term prospective studies with healthy subjects are necessary, possibly exploring both intermediate, e.g. measures of luminal narrowing, and hard outcomes. Ideally, these studies should link MNP burden in the blood, and not only in cardiovascular tissues, to CVD, in order to sustain a pathogenic role of MNPs and to explore whether the association between MNPs and CVD extends beyond patients with already manifest disease.

*In vitro* and animal studies have demonstrated the toxic potential of MPs and NPs in various cell lines and species. This experimental evidence however remains limited, and further research is needed to elucidate the physicochemical factors of MNPs on toxicity of particle size and dose on the cardiovascular system, particularly using biologically relevant exposure levels and durations.^101^ In preclinical animal models, MNPs promote oxidative stress, platelet aggregation, senescence, and inflammatory responses *in vitro* while inducing the development of atherosclerosis and several other cardiovascular alterations. However, most of these studies were conducted employing high doses of MNPs or used MNPs types with no evidence of accumulation in the human cardiovascular system. Indeed, while most preclinical studies employed PP and PS particles, evidence from *ex-vivo* samples taken from atherosclerotic plaques, thrombi, and multiple cardiac tissues suggests that PET, PA66, PE, and PVC of various size and shapes are detectable in such samples, with the latter two being prospectively associated with the incidence of CVD or mortality. Thus, preclinical studies should now be tailored to test pertinent MNP types and dosages.

Plastics are virtually ubiquitous in today's world, and thus establishing the key exposures driving their accumulation will be challenging. At present, there is no questionnaire instrument or validated laboratory procedure to assess exposure to plastics, and there are no studies exploring the associations between potential sources of exposure and MNP accumulation in tissues. Moreover, given that quantitation of MNPs in plaque samples at large scale is currently unfeasible, unless a non-invasive ad-hoc imaging method is developed, a standardized and cheap approach for MNP dosage in blood might be necessary. It is thus not yet possible to determine which, if any, MNP types are more harmful, information that could help in the implementation of mitigation or preventive measures, e.g. a reduced use for those plastics with an established pathogenic role.

To establish causality between MNPs and CVD, the accumulation of MNPs needs to be shown to precede the development of intermediate markers of atherosclerosis or other mechanisms of cardiovascular damage in a broad, non-selected population of people. Similar studies have already been conducted for other pollutants.^102^ At present, blood MNPs have not been linked to hard outcomes and, thus, specific studies are necessary. Large prospective studies collecting detailed lifestyle information coupled by serial blood sampling and monitoring the long-term incidence of hard outcomes are urgently needed to obtain a realistic picture of the relevance of the possible role of MNPs in driving CVDs. Indeed, available evidence derives from pathological contexts, e.g. from already formed atherosclerotic plaques or thrombi, impeding any speculation relative to a causal role and confining the associative evidence to patients with already manifest CVD. The knowledge available at present and relative to the possible role of MNPs in CVD is summarized in the *Graphical Abstract*.

In the event that a causal role for MNPs in the development of CVDs is established, need will emerge to develop potential preventive or therapeutic strategies. Limiting exposure to MNPs should be the preferred approach and, demonstration of a decline in CVD incidence following a reduction in plastic manufacture would further boost the argument for causality. However, the trajectory of plastic production is not likely to decline in the near-term future.^3^ Thus, beyond encouraging people to adopt behaviours limiting their personal exposure, e.g. minimizing the consumption of food and beverages packed in plastic containers, therapeutic strategies may also be envisaged. If the molecular mechanisms instigated by MNPs are confirmed, any medication counteracting such pathways might limit the deleterious consequences of MNPs. The enzymatic degradation of plastics might also be an option, similar to what has been proposed for the environment.^103^ However, none of these possible approaches has been tested for safety nor effectiveness at present, not even in animal models.

## Conclusions

The chemical exposome is increasingly recognized as a possible driver of CVD.^104^ Given the substantial residual environmental risk despite proper control of multiple risk factors, increasing levels of chemical exposures have been hypothesized as being relevant.^105,106^ While solid mechanistic and epidemiological evidence support many external pollutants, such as air pollution and some chemical exposures, there remain substantial gaps with plastics and related chemicals.^107^ Recently, a large consensus statement called for attention relatively to the possible effects of plastic pollution on health. The production and the improper disposal of plastic waste are held to impact human health at multiple levels.^3^ Plasticizer chemicals have already been linked to a range of cardiometabolic diseases,^4^ and plastic production can also affect human health and CVD development through multiple indirect routes.^3^

Recent data now suggest also MNPs as possible risk factors for CVD. Given the complexity of the topic, a multi-disciplinary effort is mandatory to gain more information relative to the role of MNPs in CVD and eventually other diseases. A large range of professional figures with diverse expertise is necessary to encompass every facet of the chain initiated by plastic-related pollution. It is easy to anticipate that the coordinated use of multiple technologies in large-scale studies and consistent economic investments through dedicated funding schemes will provide detailed and much needed information on the topic. In the meanwhile, relevant stakeholders should not ignore the already available evidence and should try to maximize the ongoing efforts aimed at reducing plastic production. This would translate into a benefit for the earth and, possibly, also for human health.

## Authors’ contributions

FP, AC, SR, PL, RM, and GP conceived the idea and wrote the manuscript. VP, RLG, LG, FO, PP, BD, PI, and MLB collected relevant literature, prepared figures and tables, provided background expertise, and critically reviewed the manuscript. The final version of the manuscript was approved by all authors.

## Supplementary data

Supplementary data are available at *European Heart Journal* online.

## Supplementary Material

ehae552_Supplementary_Data

## Declarations

### Data Availability

No data were generated or analysed for or in support of this paper.

## References

[1] https://plasticseurope.org/.

[2] Kumar M , Xiong X, He M, Tsang DCW, Gupta J, Khan E, et al Microplastics as pollutants in agricultural soils. Environ Pollut 2020;265:114980. 10.1016/j.envpol.2020.114980 32544663

[3] Landrigan PJ , Raps H, Cropper M, Bald C, Brunner M, Canonizado EM, et al The Minderoo–Monaco Commission on Plastics and Human Health. Ann Glob Health 2023;89:23. 10.5334/aogh.4056 36969097 PMC10038118

[4] Kahn LG , Philippat C, Nakayama SF, Slama R, Trasande L. Endocrine-disrupting chemicals: implications for human health. Lancet Diabetes Endocrinol 2020;8:703–18. 10.1016/S2213-8587(20)30129-7 32707118 PMC7437820

[5] Wright SL , Kelly FJ. Plastic and human health: a micro issue? Environ Sci Technol 2017;51:6634–47. 10.1021/acs.est.7b00423 28531345

[6] Zhang K , Hamidian AH, Tubic A, Zhang Y, Fang JKH, Wu C, et al Understanding plastic degradation and microplastic formation in the environment: a review. Environ Pollut 2021;274:116554. 10.1016/j.envpol.2021.116554 33529891

[7] Wong JKH , Lee KK, Tang KHD, Yap PS. Microplastics in the freshwater and terrestrial environments: prevalence, fates, impacts and sustainable solutions. Sci Total Environ 2020;719:137512. 10.1016/j.scitotenv.2020.137512 32229011

[8] Pico Y , Alfarhan A, Barcelo D. Nano- and microplastic analysis: focus on their occurrence in freshwater ecosystems and remediation technologies. TrAC Trends Anal Chem 2019;113:409–25. 10.1016/j.trac.2018.08.022

[9] Lingling Hu YZ , Wang Y, Zhang D, Pan X. Transfer of micro(nano)plastics in animals: a mini-review and future research recommendation. J Hazard Mater Adv 2022;7:100101. 10.1016/j.hazadv.2022.100101

[10] Wu P , Lin S, Cao G, Wu J, Jin H, Wang C, et al Absorption, distribution, metabolism, excretion and toxicity of microplastics in the human body and health implications. J Hazard Mater 2022;437:129361. 10.1016/j.jhazmat.2022.129361 35749897

[11] Feng Y , Tu C, Li R, Wu D, Yang J, Xia Y, et al A systematic review of the impacts of exposure to micro- and nano-plastics on human tissue accumulation and health. Eco Environ Health 2023;2:195–207. 10.1016/j.eehl.2023.08.002 38435355 PMC10902512

[12] Zhu X , Wang C, Duan X, Liang B, Genbo Xu E, Huang Z. Micro- and nanoplastics: a new cardiovascular risk factor? Environ Int 2023;171:107662. 10.1016/j.envint.2022.107662 36473237

[13] Marfella R , Prattichizzo F, Sardu C, Fulgenzi G, Graciotti L, Spadoni T, et al Microplastics and nanoplastics in atheromas and cardiovascular events. N Engl J Med 2024;390:900–10. 10.1056/NEJMoa2309822 38446676 PMC11009876

[14] Yang Y , Xie E, Du Z, Peng Z, Han Z, Li L, et al Detection of various microplastics in patients undergoing cardiac surgery. Environ Sci Technol 2023;57:10911–8. 10.1021/acs.est.2c07179 37440474

[15] Rotchell JM , Jenner LC, Chapman E, Bennett RT, Bolanle IO, Loubani M, et al Detection of microplastics in human saphenous vein tissue using muftir: a pilot study. PLoS One 2023;18:e0280594. 10.1371/journal.pone.0280594 36724150 PMC9891496

[16] Hwang J , Choi D, Han S, Jung SY, Choi J, Hong J. Potential toxicity of polystyrene microplastic particles. Sci Reps 2020;10:7391. 10.1038/s41598-020-64464-9 PMC7193629 32355311

[17] Kwon W , Kim D, Kim HY, Jeong SW, Lee SG, Kim HC, et al Microglial phagocytosis of polystyrene microplastics results in immune alteration and apoptosis in vitro and in vivo. Sci Total Environ 2022;807:150817. 10.1016/j.scitotenv.2021.150817 34627918

[18] Yan-Yang Lu HL , Ren H, Zhang X, Huang F, Zhang D, Huang Q, et al Size-dependent effects of polystyrene nanoplastics on autophagy response in human umbilical vein endothelial cells. J Hazard Mater 2022;421:126770. 10.1016/j.jhazmat.2021.126770 34358975

[19] Abihssira-García IS , Park Y, Kiron V, Olsvik PA. Fluorescent microplastic uptake by immune cells of Atlantic salmon (*Salmo salar* L.). Front Environ Sci 2020;8:560206. 10.3389/fenvs.2020.560206

[20] Florance I , Chandrasekaran N, Gopinath PM, Mukherjee A. Exposure to polystyrene nanoplastics impairs lipid metabolism in human and murine macrophages in vitro. Ecotoxicol Environ Saf 2022;238:113612. 10.1016/j.ecoenv.2022.113612 35561548

[21] Li L , Sun S, Tan L, Wang Y, Wang L, Zhang Z, et al Polystyrene nanoparticles reduced ROS and inhibited ferroptosis by triggering lysosome stress and TFEB nucleus translocation in a size-dependent manner. Nano lett 2019;19:7781–92. 10.1021/acs.nanolett.9b02795 31558022

[22] Hu M , Palic D. Micro- and nano-plastics activation of oxidative and inflammatory adverse outcome pathways. Redox Biol 2020;37:101620. 10.1016/j.redox.2020.101620 32863185 PMC7767742

[23] Tidjani A . Comparison of formation of oxidation products during photo-oxidation of linear low density polyethylene under different natural and accelerated weathering conditions. Polym Degrad Stab 2000;68:465–9. 10.1016/S0141-3910(00)00039-2

[24] Basini G , Grolli S, Bertini S, Bussolati S, Berni M, Berni P, et al Nanoplastics induced oxidative stress and VEGF production in aortic endothelial cells. Environ Toxicol Pharmacol 2023;104:104294. 10.1016/j.etap.2023.104294 37838301

[25] Vlacil AK , Banfer S, Jacob R, Trippel N, Kuzu I, Schieffer B, et al Polystyrene microplastic particles induce endothelial activation. PLoS One 2021;16:e0260181. 10.1371/journal.pone.0260181 34788346 PMC8598073

[26] Lunov O , Syrovets T, Loos C, Nienhaus GU, Mailander V, Landfester K, et al Amino-functionalized polystyrene nanoparticles activate the NLRP3 inflammasome in human macrophages. ACS Nano 2011;5:9648–57. 10.1021/nn203596e 22111911

[27] Busch M , Bredeck G, Waag F, Rahimi K, Ramachandran H, Bessel T, et al Assessing the NLRP3 inflammasome activating potential of a large panel of micro- and nanoplastics in THP-1 cells. Biomolecules 2022;12:1095. 10.3390/biom12081095 36008988 PMC9406042

[28] Caputi S , Diomede F, Lanuti P, Marconi GD, Di Carlo P, Sinjari B, et al Microplastics affect the inflammation pathway in human gingival fibroblasts: a study in the Adriatic Sea. Int J Environ Res Public Health 2022;19:7782. 10.3390/ijerph19137782 35805437 PMC9266176

[29] Alijagic A , Hedbrant A, Persson A, Larsson M, Engwall M, Sarndahl E. NLRP3 inflammasome as a sensor of micro- and nanoplastics immunotoxicity. Front Immunol 2023;14:1178434. 10.3389/fimmu.2023.1178434 37143682 PMC10151538

[30] Yang W , Jannatun N, Zeng Y, Liu T, Zhang G, Chen C, et al Impacts of microplastics on immunity. Front Toxicol 2022;4:956885. 10.3389/ftox.2022.956885 36238600 PMC9552327

[31] Swanson KV , Deng M, Ting JPY. The NLRP3 inflammasome: molecular activation and regulation to therapeutics. Nat Rev Immunol 2019;19:477–89. 10.1038/s41577-019-0165-0 31036962 PMC7807242

[32] Wiley CD , Campisi J. The metabolic roots of senescence: mechanisms and opportunities for intervention. Nat Metab 2021;3:1290–301. 10.1038/s42255-021-00483-8 34663974 PMC8889622

[33] Jin W , Zhang W, Tang H, Wang P, Zhang Y, Liu S, et al Microplastics exposure causes the senescence of human lung epithelial cells and mouse lungs by inducing ROS signaling. Environ Int 2024;185:108489. 10.1016/j.envint.2024.108489 38367553

[34] Dhakal B , Shiwakoti S, Park EY, Kang KW, Schini-Kerth VB, Park SH, et al SGLT2 inhibition ameliorates nano plastics-induced premature endothelial senescence and dysfunction. Sci Rep 2023;13:6256. 10.1038/s41598-023-33086-2 37069192 PMC10110533

[35] Shiwakoti S , Ko JY, Gong D, Dhakal B, Lee JH, Adhikari R, et al Effects of polystyrene nanoplastics on endothelium senescence and its underlying mechanism. Environ Int 2022;164:107248. 10.1016/j.envint.2022.107248 35461096

[36] Birch J , Gil J. Senescence and the SASP: many therapeutic avenues. Genes Dev 2020;34:1565–76. 10.1101/gad.343129.120 33262144 PMC7706700

[37] McGuire DK , Shih WJ, Cosentino F, Charbonnel B, Cherney DZI, Dagogo-Jack S, et al Association of SGLT2 inhibitors with cardiovascular and kidney outcomes in patients with type 2 diabetes: a meta-analysis. JAMA Cardiol 2021;6:148–58. 10.1001/jamacardio.2020.4511 33031522 PMC7542529

[38] Marfella R , Sardu C, 'Onofrio D, Fumagalli N, Scisciola C, Sasso L, et al SGLT-2 inhibitors and in-stent restenosis-related events after acute myocardial infarction: an observational study in patients with type 2 diabetes. BMC Med 2023;21:71. 10.1186/s12916-023-02781-2 36829203 PMC9960194

[39] Ceriello A , Lucisano G, Prattichizzo F, La Grotta R, Frige C, De Cosmo S, et al The legacy effect of hyperglycemia and early use of SGLT-2 inhibitors: a cohort study with newly-diagnosed people with type 2 diabetes. Lancet Reg Health Eur 2023;31:100666. 10.1016/j.lanepe.2023.100666 37547276 PMC10398589

[40] Barshtein G , Arbell D, Yedgar S. Hemolytic effect of polymeric nanoparticles: role of albumin. IEEE Trans Nanobioscience 2011;10:259–61. 10.1109/TNB.2011.2175745 22128012

[41] McGuinnes C , Duffin R, Brown S, L Mills N, Megson IL, Macnee W, et al Surface derivatization state of polystyrene latex nanoparticles determines both their potency and their mechanism of causing human platelet aggregation in vitro. Toxicol Sci 2011;119:359–68. 10.1093/toxsci/kfq349 21123846

[42] Oslakovic C , Cedervall T, Linse S, Dahlback B. Polystyrene nanoparticles affecting blood coagulation. Nanomedicine 2012;8:981–6. 10.1016/j.nano.2011.12.001 22197724

[43] Parkinson SJ , Tungsirisurp S, Joshi C, Richmond BL, Gifford ML, Sikder A, et al Polymer nanoparticles pass the plant interface. Nat Commun 2022;13:7385. 10.1038/s41467-022-35066-y 36450796 PMC9712430

[44] Kenesei K , Murali K, Czeh A, Piella J, Puntes V, Madarasz E. Enhanced detection with spectral imaging fluorescence microscopy reveals tissue- and cell-type-specific compartmentalization of surface-modified polystyrene nanoparticles. J Nanobiotechnology 2016;14:55. 10.1186/s12951-016-0210-0 27388915 PMC4936314

[45] Liang B , Zhong Y, Huang Y, Lin X, Liu J, Lin L, et al Underestimated health risks: polystyrene micro- and nanoplastics jointly induce intestinal barrier dysfunction by ROS-mediated epithelial cell apoptosis. Part Fibre Toxicol 2021;18:20. 10.1186/s12989-021-00414-1 34098985 PMC8186235

[46] Jasinski J , Volkl M, Hahn J, Jerome V, Freitag R, Scheibel T. Polystyrene microparticle distribution after ingestion by murine macrophages. J Hazard Mater 2023;457:131796. 10.1016/j.jhazmat.2023.131796 37307726

[47] Walczak AP , Hendriksen PJ, Woutersen RA, van der Zande M, Undas AK, Helsdingen R, et al Bioavailability and biodistribution of differently charged polystyrene nanoparticles upon oral exposure in rats. J Nanopart Res 2015;17:231. 10.1007/s11051-015-3029-y 26028989 PMC4440892

[48] Stock V , Bohmert L, Lisicki E, Block R, Cara-Carmona J, Pack LK, et al Uptake and effects of orally ingested polystyrene microplastic particles in vitro and in vivo. Arch Toxicol 2019;93:1817–33. 10.1007/s00204-019-02478-7 31139862

[49] Smyth E , Solomon A, Vydyanath A, Luther PK, Pitchford S, Tetley TD, et al Induction and enhancement of platelet aggregation in vitro and in vivo by model polystyrene nanoparticles. Nanotoxicology 2015;9:356–64. 10.3109/17435390.2014.933902 25030098

[50] Enyoh CE , Shafea L, Verla AW, Verla EN, Qingyue W, Chowdhury T, et al Microplastics exposure routes and toxicity studies to ecosystems: an overview. Environ Anal Health Toxicol 2020;35:e2020004. 10.5620/eaht.e2020004 32570999 PMC7308665

[51] da Silva Brito WA , Mutter F, Wende K, Cecchini AL, Schmidt A, Bekeschus S. Consequences of nano and microplastic exposure in rodent models: the known and unknown. Part Fibre Toxicol 2022;19:28. 10.1186/s12989-022-00473-y 35449034 PMC9027452

[52] https://www.who.int/publications/i/item/9789241516198.

[53] Nemmar A , Hoylaerts MF, Hoet PH, Vermylen J, Nemery B. Size effect of intratracheally instilled particles on pulmonary inflammation and vascular thrombosis. Toxicol Appl Pharmacol 2003;186:38–45. 10.1016/S0041-008X(02)00024-8 12583991

[54] Zhang M , Shi J, Zhu Y, Pan H, Song L, Deng H. Polystyrene nanoplastics induce vascular stenosis via regulation of the PIWI-interacting RNA expression profile. Environ Pollut 2024;345:123441. 10.1016/j.envpol.2024.123441 38272162

[55] Silva VM , Corson N, Elder A, Oberdorster G. The rat ear vein model for investigating in vivo thrombogenicity of ultrafine particles (UFP). Toxicol Sci 2005;85:983–9. 10.1093/toxsci/kfi142 15772370

[56] Bihari P , Holzer M, Praetner M, Fent J, Lerchenberger M, Reichel CA, et al Single-walled carbon nanotubes activate platelets and accelerate thrombus formation in the microcirculation. Toxicology 2010;269:148–54. 10.1016/j.tox.2009.08.011 19698757

[57] Li Z , Zhu S, Liu Q, Wei J, Jin Y, Wang X, et al Polystyrene microplastics cause cardiac fibrosis by activating Wnt/beta-catenin signaling pathway and promoting cardiomyocyte apoptosis in rats. Environ Pollut 2020;265:115025. 10.1016/j.envpol.2020.115025 32806417

[58] Roshanzadeh A , Oyunbaatar NE, Ganjbakhsh SE, Park S, Kim DS, Kanade PP, et al Exposure to nanoplastics impairs collective contractility of neonatal cardiomyocytes under electrical synchronization. Biomaterials 2021;278:121175. 10.1016/j.biomaterials.2021.121175 34628193

[59] Wei J , Wang X, Liu Q, Zhou N, Zhu S, Li Z, et al The impact of polystyrene microplastics on cardiomyocytes pyroptosis through NLRP3/caspase-1 signaling pathway and oxidative stress in wistar rats. Environ Toxicol 2021;36:935–44. 10.1002/tox.23095 33404188

[60] Zhao J , Gomes Dl, Jin L, Mathis SP, Li X, Rouchka EC, et al Polystyrene bead ingestion promotes adiposity and cardiometabolic disease in mice. Ecotoxicol Environ Saf 2022;232:113239. 10.1016/j.ecoenv.2022.113239 35093814 PMC8860873

[61] Yan J , Pan Y, He J, Pang X, Shao W, Wang C, et al Toxic vascular effects of polystyrene microplastic exposure. Sci Total Environ 2023;905:167215. 10.1016/j.scitotenv.2023.167215 37734602

[62] Zhang T , Yang S, Ge Y, Wan X, Zhu Y, Yang F, et al Multi-dimensional evaluation of cardiotoxicity in mice following respiratory exposure to polystyrene nanoplastics. Part Fibre Toxicol 2023;20:46. 10.1186/s12989-023-00557-3 38031128 PMC10685678

[63] Zhou Y , Wu Q, Li Y, Feng Y, Wang Y, Cheng W. Low-dose of polystyrene microplastics induce cardiotoxicity in mice and human-originated cardiac organoids. Environ Int 2023;179:108171. 10.1016/j.envint.2023.108171 37669592

[64] Zhang M , Shi J, Zhou J, Song L, Ding J, Deng HP, et al N6-methyladenosine methylation mediates non-coding RNAs modification in microplastic-induced cardiac injury. Ecotoxicol Environ Saf 2023;262:115174. 10.1016/j.ecoenv.2023.115174 37354568

[65] Wang B , Liang B, Huang Y, Li Z, Zhang B, Du J, et al Long-chain acyl carnitines aggravate polystyrene nanoplastics-induced atherosclerosis by upregulating marco. Adv Sci 2023;10:e2205876. 10.1002/advs.202205876 PMC10323628 37144527

[66] Zhang M , Shi J, Pan H, Zhu J, Wang X, Song L, et al A novel tiRNA-Glu-CTC induces nanoplastics accelerated vascular smooth muscle cell phenotypic switching and vascular injury through mitochondrial damage. Sci Total Environ 2024;912:169515. 10.1016/j.scitotenv.2023.169515 38154651

[67] Liu Z , Zhuan Q, Zhang L, Meng L, Fu X, Hou Y. Polystyrene microplastics induced female reproductive toxicity in mice. J Hazard Mater 2022;424:127629. 10.1016/j.jhazmat.2021.127629 34740508

[68] Horton AA , Walton A, Spurgeon DJ, Lahive E, Svendsen C. Microplastics in freshwater and terrestrial environments: evaluating the current understanding to identify the knowledge gaps and future research priorities. Sci Total Environ 2017;586:127–41. 10.1016/j.scitotenv.2017.01.190 28169032

[69] Prata JC . Airborne microplastics: consequences to human health? Environ Pollut 2018;234:115–26. 10.1016/j.envpol.2017.11.043 29172041

[70] Lofty J , Muhawenimana V, Wilson C, Ouro P. Microplastics removal from a primary settler tank in a wastewater treatment plant and estimations of contamination onto European agricultural land via sewage sludge recycling. Environ Pollut 2022;304:119198. 10.1016/j.envpol.2022.119198 35341817

[71] Gan Q , Cui J, Jin B. Environmental microplastics: classification, sources, fates, and effects on plants. Chemosphere 2023;313:137559. 10.1016/j.chemosphere.2022.137559 36528162

[72] Cox KD , Covernton GA, Davies HL, Dower JF, Juanes F, Dudas SE. Human consumption of microplastics. Environ Sci Technol 2019;53:7068–74. 10.1021/acs.est.9b01517 31184127

[73] Kannan K , Vimalkumar K. A review of human exposure to microplastics and insights into microplastics as obesogens. Front Endocrinol 2021;12:724989. 10.3389/fendo.2021.724989 PMC8416353 34484127

[74] Mohamed Nor NH , Kooi M, Diepens NJ, Koelmans AA. Lifetime accumulation of microplastic in children and adults. Environ Sci Technol 2021;55:5084–96. 10.1021/acs.est.0c07384 33724830 PMC8154366

[75] Senathirajah K , Attwood S, Bhagwat G, Carbery M, Wilson S, Palanisami T. Estimation of the mass of microplastics ingested—a pivotal first step towards human health risk assessment. J Hazard Mater 2021;404:124004. 10.1016/j.jhazmat.2020.124004 33130380

[76] Ragusa A , Svelato A, Santacroce C, Catalano P, Notarstefano V, Carnevali O, et al Plasticenta: first evidence of microplastics in human placenta. Environ Int 2021;146:106274. 10.1016/j.envint.2020.106274 33395930

[77] Braun T , Ehrlich L, Henrich W, Koeppel S, Lomako I, Schwabl P, et al Detection of microplastic in human placenta and meconium in a clinical setting. Pharmaceutics 2021;13:921. 10.3390/pharmaceutics14010013 34206212 PMC8308544

[78] Liu S , Guo J, Liu X, Yang R, Wang H, Sun Y, et al Detection of various microplastics in placentas, meconium, infant feces, breastmilk and infant formula: a pilot prospective study. Sci Total Environ 2023;854:158699. 10.1016/j.scitotenv.2022.158699 36108868

[79] Zhu L , Zhu J, Zuo R, Xu Q, Qian Y, An L. Identification of microplastics in human placenta using laser direct infrared spectroscopy. Sci Total Environ 2023;856:159060. 10.1016/j.scitotenv.2022.159060 36174702

[80] Jenner LC , Rotchell JM, Bennett RT, Cowen M, Tentzeris V, Sadofsky LR. Detection of microplastics in human lung tissue using muftir spectroscopy. Sci Total Environ 2022;831:154907. 10.1016/j.scitotenv.2022.154907 35364151

[81] Amato-Lourenco LF , Carvalho-Oliveira R, Junior GR, Dos Santos Galvao L, Ando RA, Mauad T. Presence of airborne microplastics in human lung tissue. J Hazard Mater 2021;416:126124. 10.1016/j.jhazmat.2021.126124 34492918

[82] Chen Q , Gao J, Yu H, Su H, Yang Y, Cao Y, et al An emerging role of microplastics in the etiology of lung ground glass nodules. Environ Sci Eur 2022;34:25. 10.1186/s12302-022-00605-3

[83] Horvatits T , Tamminga M, Liu B, Sebode M, Carambia A, Fischer L, et al Microplastics detected in cirrhotic liver tissue. EBioMedicine 2022;82:104147. 10.1016/j.ebiom.2022.104147 35835713 PMC9386716

[84] Ragusa A , Notarstefano V, Svelato A, Belloni A, Gioacchini G, Blondeel C, et al Raman microspectroscopy detection and characterisation of microplastics in human breastmilk. Polymers (Basel) 2022;14:2700. 10.3390/polym14132700 35808745 PMC9269371

[85] Pironti C , Notarstefano V, Ricciardi M, Motta O, Giorgini E, Montano L. First evidence of microplastics in human urine, a preliminary study of intake in the human body. Toxics 2022;11:40. 10.3390/toxics11010040 36668766 PMC9867291

[86] Huang S , Huang X, Bi R, Guo Q, Yu X, Zeng Q, et al Detection and analysis of microplastics in human sputum. Environ Sci Technol 2022;56:2476–86. 10.1021/acs.est.1c03859 35073488

[87] Li Z , Wang J, Gao X, Du J, Sui H, Wu J, et al Investigation of microplastics (≥10 mum) in meconium by Fourier transform infrared microspectroscopy. Toxics 2023;11:310. 10.3390/toxics11040310 37112537 PMC10143218

[88] Zhang J , Wang L, Trasande L, Kannan K. Occurrence of polyethylene terephthalate and polycarbonate microplastics in infant and adult feces. Environ Sci Technol Lett 2021;8:989–94. 10.1021/acs.estlett.1c00559

[89] Yan Z , Liu Y, Zhang T, Zhang F, Ren H, Zhang Y. Analysis of microplastics in human feces reveals a correlation between fecal microplastics and inflammatory bowel disease status. Environ Sci Technol 2022;56:414–21. 10.1021/acs.est.1c03924 34935363

[90] Leslie HA , van Velzen MJM, Brandsma SH, Vethaak AD, Garcia-Vallejo JJ, Lamoree MH. Discovery and quantification of plastic particle pollution in human blood. Environ Int 2022;163:107199. 10.1016/j.envint.2022.107199 35367073

[91] Massardo S , Verzola D, Alberti S, Caboni C, Santostefano M, Eugenio Verrina E, et al MicroRaman spectroscopy detects the presence of microplastics in human urine and kidney tissue. Environ Int 2024;184:108444. 10.1016/j.envint.2024.108444 38281449

[92] Ibrahim YS , Tuan Anuar S, Azmi AA, Wan Mohd Khalik WMA, Lehata S, Hamzah SR, et al Detection of microplastics in human colectomy specimens. JGH Open 2021;5:116–21. 10.1002/jgh3.12457 33490620 PMC7812470

[93] Montano L , Giorgini E, Notarstefano V, Notari T, Ricciardi M, Piscopo M, et al Raman microspectroscopy evidence of microplastics in human semen. Sci Total Environ 2023;901:165922. 10.1016/j.scitotenv.2023.165922 37532047

[94] Sun J , Sui M, Wang T, Teng X, Sun J, Chen M. Detection and quantification of various microplastics in human endometrium based on laser direct infrared spectroscopy. Sci Total Environ 2024;906:167760. 10.1016/j.scitotenv.2023.167760 37832687

[95] Zhu L , Kang Y, Ma M, Wu Z, Zhang L, Hu R, et al Tissue accumulation of microplastics and potential health risks in human. Sci Total Environ 2024;915:170004. 10.1016/j.scitotenv.2024.170004 38220018

[96] Wu D , Feng Y, Wang R, Jiang J, Guan Q, Yang X, et al Pigment microparticles and microplastics found in human thrombi based on Raman spectral evidence. J Adv Res 2023;49:141–50. 10.1016/j.jare.2022.09.004 36116710 PMC10334115

[97] Wang T , Yi Z, Liu X, Cai Y, Huang X, Fang J, et al Multimodal detection and analysis of microplastics in human thrombi from multiple anatomically distinct sites. EBioMedicine 2024;103:105118. 10.1016/j.ebiom.2024.105118 38614011 PMC11021838

[98] Liu S , Wang C, Yang Y, Du Z, Li L, Zhang M, et al Microplastics in three types of human arteries detected by pyrolysis–gas chromatography/mass spectrometry (Py–GC/MS). J Hazard Mater 2024;469:133855. 10.1016/j.jhazmat.2024.133855 38428296

[99] Campen M , Nihart A, Garcia M, Liu R, Olewine M, Castillo E, et al Bioaccumulation of microplastics in decedent human brains assessed by pyrolysis gas chromatography–mass spectrometry. Res Squ 2024;6:rs.3.rs-4345687. 10.21203/rs.3.rs-4345687/v1

[100] Thornton Hampton LM , Bouwmeester H, Brander SM, Coffin S, Cole M, Hermabessiere L, et al Research recommendations to better understand the potential health impacts of microplastics to humans and aquatic ecosystems. Microplast Nanoplast 2022;2:18. 10.1186/s43591-022-00038-y

[101] Gonzalez-Acedo A , Garcia-Recio E, Illescas-Montes R, Ramos-Torrecillas J, Melguizo-Rodriguez L, Costela-Ruiz VJ. Evidence from in vitro and in vivo studies on the potential health repercussions of micro- and nanoplastics. Chemosphere 2021;280:130826. 10.1016/j.chemosphere.2021.130826 34162123

[102] Abohashem S , Osborne MT, Dar T, Naddaf N, Abbasi T, Ghoneem A, et al A leucopoietic–arterial axis underlying the link between ambient air pollution and cardiovascular disease in humans. Eur Heart J 2021;42:761–72. 10.1093/eurheartj/ehaa982 33428721 PMC7882372

[103] Kaushal J , Khatri M, Arya SK. Recent insight into enzymatic degradation of plastics prevalent in the environment: a mini-review. Clean Eng Technol 2021;2:100083. 10.1016/j.clet.2021.100083

[104] Montone RA , Camilli M, Calvieri C, Magnani G, Bonanni A, Bhatt DL, et al Exposome in ischaemic heart disease: beyond traditional risk factors. Eur Heart J 2024;45:419–38. 10.1093/eurheartj/ehae001 38238478 PMC10849374

[105] Crea F . Focus on residual cardiovascular risk: air pollution, infections, socioeconomic status, and lipopoprotein(a). Eur Heart J 2024;45:971–5. 10.1093/eurheartj/ehae160 38538150

[106] Al-Kindi S , Paneni F, Brook RD, Rajagopalan S. Residual environmental risk in patients with cardiovascular disease: an overlooked paradigm. Eur Heart J 2023;44:4612–4. 10.1093/eurheartj/ehad412 37431798 PMC10659939

[107] Rajagopalan S , Landrigan PJ. Pollution and the heart. N Engl J Med 2021;385:1881–92. 10.1056/NEJMra2030281 34758254

