# Supplementary material for: Micro-nanoplastics and cardiovascular diseases: evidence and perspectives
Source: Eur Heart J. 2024 Sep 6;45(38):4099–110. doi: 10.1093/eurheartj/ehae552 (PMC11458152; doi:10.1093/eurheartj/ehae552)
Supplement: ehae552_Supplementary_Data [file ehae552_supplementary_data.pdf]

SUPPLEMENTARY DATA

Supplement to: Prattichizzo F, et al. Micro-Nanoplastics and Cardiovascular Diseases: Evidence and Perspectives

Prattichizzo F, et al.

TABLE OF CONTENTS

1. **Supplementary Figure 1.** Evidence of micro- and nano-plastics accumulation in human tissues or biological fluids, excluding the cardiovascular system……………………………1
2. **Supplementary Table 1**. Summary of the studies assessing the effect of micro-nanoplastics in *in vitro* models in the context of cardiovascular diseases…………………………………3
3. **References for Supplementary Table 1**. …………………………………………………..5

Supplementary Figure S1. Evidence of micro- and nano-plastics accumulation in human tissues or biological fluids, excluding the cardiovascular system.

Lung

Technique	µ-FTIR, µRaman spectroscopy
Plastic Type	12 polymers (PET and PP the most abundant)
Size	4-88 µm

Sputum

Technique	µ-FTIR
Plastic Type	21 types of microplastics of which the most prevalent were PU and PES
Size	< 500 µm (median: 75.43 µm; interquartile range: 44.67–210.64 µm)

Semen and testis

Technique	Py-GC/MS, LD-IR
Plastic Type	PS (testis), PE and PVC (semen)
Dimension	20–100 µm
Mean Concentration	0.23 ± 0.45 particles/mL in semen and 11.60 ± 15.52 particles/g in testis

Blood

Technique	Py-GC/MS
Plastic Type	PMMA, PP, PS, PE, PET
Mean Concentration	1.6 µg/mL

Meconium, infant and adult stool

Technique	HPLC–MS/MS analysis, µRaman spectroscopy
Plastic Type	PET, PC, TPA, BPA, PA, PP, PE
Mean Concentration in meconium	PET (12,000 and 3200 ng/g); PC (110 ng/g dw)
Mean Concentration in infants stool	PET (5700–82,000 ng/g); PC (49–2100 ng/g)
Mean Concentration in healthy adults stool	PET (2200–16,000 ng/g); PC (37–620 ng/g)
Size in healthy and IBD patients' stool	Healthy: 4.4−333.2 µm<br>IBD: 1.7−393.8 µm

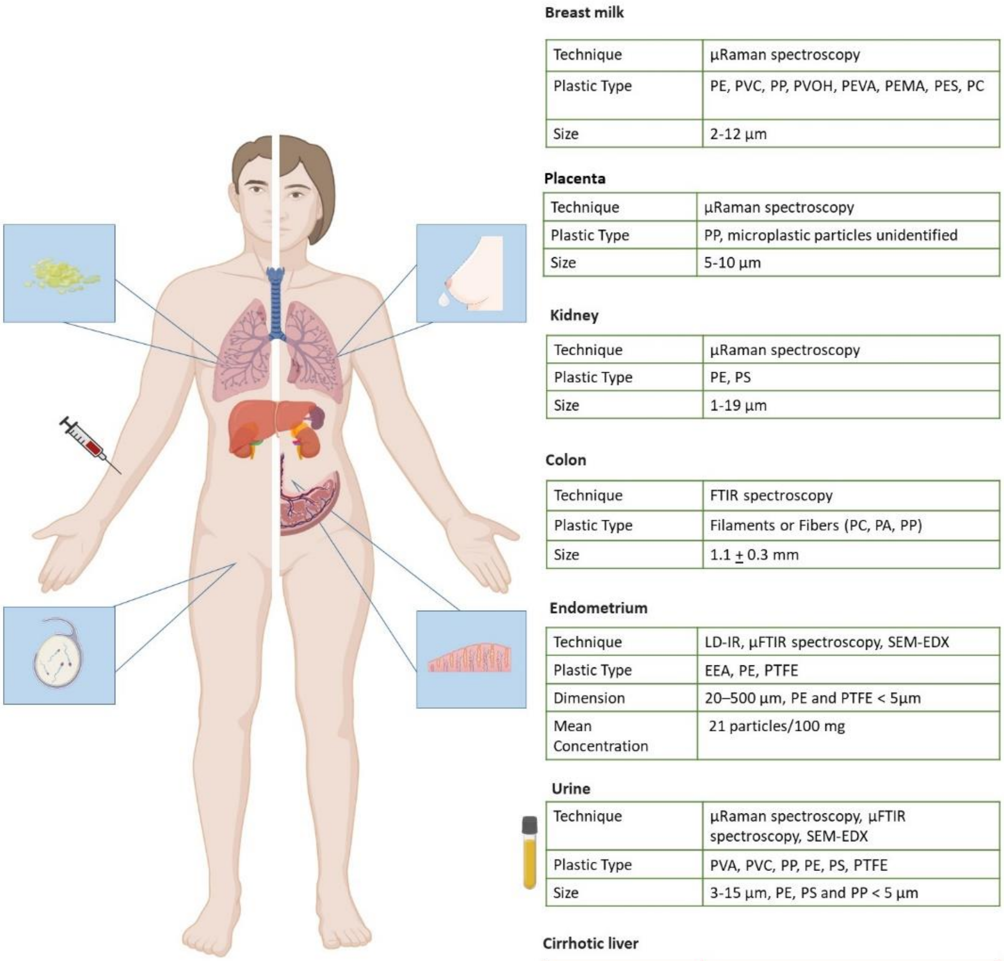

Breast milk

Technique	µRaman spectroscopy
Plastic Type	PE, PVC, PP, PVOH, PEVA, PEMA, PES, PC
Size	2-12 µm

Placenta

Technique	µRaman spectroscopy
Plastic Type	PP, microplastic particles unidentified
Size	5-10 µm

Kidney

Technique	µRaman spectroscopy
Plastic Type	PE, PS
Size	1-19 µm

Colon

Technique	FTIR spectroscopy
Plastic Type	Filaments or Fibers (PC, PA, PP)
Size	1.1 ± 0.3 mm

Endometrium

Technique	LD-IR, µFTIR spectroscopy, SEM-EDX
Plastic Type	EEA, PE, PTFE
Dimension	20–500 µm, PE and PTFE < 5µm
Mean Concentration	21 particles/100 mg

Urine

Technique	µRaman spectroscopy, µFTIR spectroscopy, SEM-EDX
Plastic Type	PVA, PVC, PP, PE, PS, PTFE
Size	3-15 µm, PE, PS and PP < 5 µm

Cirrhotic liver

Technique	µRaman spectroscopy
Plastic Type	PS, PVC, PET, PMMA, POM, PP
Size	From 4 to 30 µm

Summary of the evidence relative to the presence of different micro-nanoplastics (MNP)s in various organs, excluding the cardiovascular system, along with the technology used for their detection, the size, or the concentrations of the different MNPs. Red boxes evidence the only results showing a cross-sectional association with a disease.

List of acronyms**:** PC: polycarbonate; µ-FTIR: micro Fourier Transform Interferometer spectroscopy; LDPE, low-density polyethylene; PET: Polyethylene terephthalate; PP: Polypropylene; PS: Polystyrene; PU: Polyurethane; PVC: Polyvinyl chloride; LD-IR: Laser direct infrared spectroscopy; PA, polyamide; PTFE: polytetrafluoroethylene; PAN: poliacrilonitrile; PES: Polietersulfone; PVA, polyvinyl alcohol; TPA: Thermoplastic polyamide elastomers; BPA: Bisphenol A; PVOH: Polyvinyl

Alcohol; PEVA, polyethylene vinyl acetate; PEMA Poly Ethyl Methacrylate; EEA: Ethylene/ethylacrylate; PMMA: Polymethyl Methacrylate; POM: polyoxymethylene; HPLC–HRMS, high performance liquid chromatography–high-resolution mass spectrometry; Py-GC/MS: Pyrolysis–gas chromatography–mass spectrometry; IBD: Inflammatory Bowel Disease.

Supplementary Table S1. Summary of the studies assessing the effect of micro-nanoplastics in *in vitro* models in the context of cardiovascular diseases.

Study	Cell model	Micro-nanoplastic type (and size)	Dose	Treatment	Condition studied	Effects observed
McGuinnes C, et al., 2011[1]	Human venous blood	Polystyrene latex nanoparticles (PLNP), unmodified (umPLNP), aminated (aPLNP), and carboxylated (cPLNP) (50 nm)	200 to 1000 µg/mL	Erythrocytes are incubated with MNPs for 20 min	Platelet aggregation	Amine-PLNPs cause significant hemolysis, abolished by the presence of 5% plasma. Both the carboxylate- and amine-PLNPs cause platelet aggregation; platelets treated with carboxylate-PLNPs exhibit increased CD62P and PAC-1 expression.
Barshtein G, et al., 2011[2]	Human red blood cell suspension	PS (50, 107, and 250 nm)	50, 150, 250, 350, and 500 µg/mL	Supplemented with 0.01–0.5% albumin in PBS buffer for 1 hour	Hemolysis	MNPs induced hemolysis. This effect is inhibited by the presence of albumin and larger particles
Oslakovic C, et al., 2012[3]	Human plasma	Polystyrene (PS) (23, 24, 57, 200, 220, and 330 nm)	0.06, 0.13, 0.25, and 0.5 mg/mL	Plasma is incubated with MNPs	Blood coagulation	Amine-PS MNPs downregulate thrombin with a major effect of smaller MNPs
Barshtein G, et al., 2016[4]	Human red blood cells and bone marrow endothelial cells	PS (about 50, 107, 250 nm)	0.05, 0.2, and 0.5 mg/ mL	Cell suspension is incubated with MNPs, for 1 hour	Aggregation of red blood cells and their adhesion to endothelial cells	Activate red blood cells aggregation and adhesion to endothelial cells
Bojic et al., 2020[5]	Human induced pluripotent stem cells and early human expanded blastocysts	PS (40 and 200 nm)	1 × 10^9 items/mL	24 hours	Development of atrioventricular heart valves; cellular components	MNPs cause abnormalities in the development of the atrioventricular valve and dysfunction in the extracellular matrix.

Hwang J, et al., 2020[6]	Human peripheral blood mononuclear cells	PS (0.46, 1, 3, 10, 40, and 100 µm)	0.5, 1, 10, 100, 500, and 1,000 µg/mL	*In-vivo* and *in-vitro* hemolysis assays	Immune response and inflammation	MNPs smaller than 5 µm in diameter exerted hemolytic effects (approximately 4%)
Vlacil AK, et al., 2021[7]	Murine myocardial endothelial cells and monocytic J774A.1 cells	PS (1 µm)	0.54, 54, and 5,400 ng/ mL	Under static and flow conditions for 3 and 6 hours	Inflammation	Carboxylate-PS-MNPs induced the expression of Vcam-1 and Icam-1 in ECs, and IL-1β and TNFα in monocytic cells.
Lee HS, et al., 2021[8]	Human umbilical vein endothelial cells	PS (0.5, 1, and 5 µm)	0, 20, 40, 60, 80, and 100 µg/mL	24, 48, 72 hours	Angiogenic signaling pathways, wound healing, cell migration, autophagic and necrotic cell death.	After 6 hours of exposure to PS-MNPs, endothelial cells showed reduced tube-forming capacity, while exposure for 48 hours results in cytotoxicity
Lu et al., 2022[9]	Human umbilical vein endothelial cells	PS (100 and 500 nm)	0, 5, 10, 25, 50, and 100 µg/mL	Cell suspension is incubated with MNPs for 48 hours	Autophagy	MNPs (100 and 500 nm) caused cell membrane damage
Shiwakoti S, et al., 2022[10]	Pulmonary microvascular endothelial cells	PS (25 nm)	0.1, 1, and 10 µg/mL	Incubated with PS	Senescence	MNPs induced premature senescence and endothelial dysfunction
Basini G, et al., 2023[11]	Immortalized porcine aortic endothelial cell line	Fluorescent PS (100 nm diameter)	5, 25 and 75 µg//mL	48h treatment	VEGF expression and production, redox status	MNPs promoted a disruption both in metabolic activity and redox status in endothelial cell
Zhou Y, et al., 2023[12]	Human-originated cardiac organoids	PS (1 µm)	0.025, 0.25 and 2.5 µg/mL	On day 18, 72h treatment	Oxidative stress, inflammatory response, and apoptosis.	MNPs induced cardiac hypertrophy both *in vivo* and *in vitro* experiments

References for Supplementary Table 1

1. McGuinnes C, Duffin R, Brown S, N LM, Megson IL, Macnee W, et al. Surface derivatization state of polystyrene latex nanoparticles determines both their potency and their mechanism of causing human platelet aggregation in vitro. *Toxicological sciences : an official journal of the Society of Toxicology*. 2011;119:359-368
2. Barshtein G, Arbell D, Yedgar S. Hemolytic effect of polymeric nanoparticles: Role of albumin. *IEEE transactions on nanobioscience*. 2011;10:259-261
3. Oslakovic C, Cedervall T, Linse S, Dahlback B. Polystyrene nanoparticles affecting blood coagulation. *Nanomedicine : nanotechnology, biology, and medicine*. 2012;8:981-986
4. Barshtein G, Livshits L, Shvartsman LD, Shlomai NO, Yedgar S, Arbell D. Polystyrene nanoparticles activate erythrocyte aggregation and adhesion to endothelial cells. *Cell biochemistry and biophysics*. 2016;74:19-27
5. Bojic S, Falco MM, Stojkovic P, Ljujic B, Gazdic Jankovic M, Armstrong L, et al. Platform to study intracellular polystyrene nanoplastic pollution and clinical outcomes. *Stem cells*. 2020;38:1321-1325
6. Hwang J, Choi D, Han S, Jung SY, Choi J, Hong J. Potential toxicity of polystyrene microplastic particles. *Scientific reports*. 2020;10:7391
7. Vlacil AK, Banfer S, Jacob R, Trippel N, Kuzu I, Schieffer B, et al. Polystyrene microplastic particles induce endothelial activation. *PloS one*. 2021;16:e0260181
8. Lee HS, Amarakoon D, Wei CI, Choi KY, Smolensky D, Lee SH. Adverse effect of polystyrene microplastics (ps-mps) on tube formation and viability of human umbilical vein endothelial cells. *Food and chemical toxicology : an international journal published for the British Industrial Biological Research Association*. 2021;154:112356
9. Lu YY, Li H, Ren H, Zhang X, Huang F, Zhang D, et al. Size-dependent effects of polystyrene nanoplastics on autophagy response in human umbilical vein endothelial cells. *Journal of hazardous materials*. 2022;421:126770
10. Shiwakoti S, Ko JY, Gong D, Dhakal B, Lee JH, Adhikari R, et al. Effects of polystyrene nanoplastics on endothelium senescence and its underlying mechanism. *Environment international*. 2022;164:107248
11. Basini G, Grolli S, Bertini S, Bussolati S, Berni M, Berni P, et al. Nanoplastics induced oxidative stress and vegf production in aortic endothelial cells. *Environmental toxicology and pharmacology*. 2023;104:104294
12. Zhou Y, Wu Q, Li Y, Feng Y, Wang Y, Cheng W. Low-dose of polystyrene microplastics induce cardiotoxicity in mice and human-originated cardiac organoids. *Environment international*. 2023;179:108171
